# Expanded alternative splice isoform profiling of the mouse Ca_v_3.1/α1G T-type calcium channel

**DOI:** 10.1186/1471-2199-10-53

**Published:** 2009-05-29

**Authors:** Wayne L Ernst, Jeffrey L Noebels

**Affiliations:** 1 Developmental Neurogenetics Laboratory, Baylor College of Medicine, Houston, TX 77030, USA; 2 Department of Molecular & Human Genetics, Baylor College of Medicine, Houston, TX 77030, USA; 3 Department of Neurology, Baylor College of Medicine, Houston, TX 77030, USA; 4 Department of Neuroscience, Baylor College of Medicine, Houston, TX 77030, USA

## Abstract

**Background:**

Alternative splicing of low-voltage-activated T-type calcium channels contributes to the molecular and functional diversity mediating complex network oscillations in the normal brain. Transcript scanning of the human *CACNA1G *gene has revealed the presence of 11 regions within the coding sequence subjected to alternative splicing, some of which enhance T-type current. In mouse models of absence epilepsy, elevated T-type calcium currents without clear increases in channel expression are found in thalamic neurons that promote abnormal neuronal synchronization. To test whether enhanced T-type currents in these models reflect pathogenic alterations in channel splice isoforms, we determined the extent of alternative splicing of mouse *Cacna1g *transcripts and whether evidence of altered transcript splicing could be detected in mouse absence epilepsy models.

**Results:**

Transcript scanning of the murine *Cacna1g *gene detected 12 regions encoding alternative splice isoforms of Ca_v_3.1/α1G T-type calcium channels. Of the 12 splice sites, six displayed homology to the human *CACNA1G *splice sites, while six novel mouse-specific splicing events were identified, including one intron retention, three alternative acceptor sites, one alternative donor site, and one exon exclusion. In addition, two brain region-specific alternative splice patterns were observed in the cerebellum. Comparative analyses of brain regions from four monogenic absence epilepsy mouse models with altered thalamic T-type currents and wildtype controls failed to reveal differences in *Cacna1g *splicing patterns.

**Conclusion:**

The determination of six novel alternative splice sites within the coding region of the mouse *Cacna1g *gene greatly expands the potential biophysical diversity of voltage-gated T-type channels in the mouse central nervous system. Although alternative splicing of Ca_v_3.1/α1G channels does not explain the enhancement of T-type current identified in four mouse models of absence epilepsy, post-transcriptional modification of T-type channels through this mechanism may influence other developmental neurological phenotypes.

## Background

Alternative splicing of pre-mRNAs is a critical regulatory mechanism augmenting mRNA transcript diversity and expanding protein function [1]. Within the nervous system, alternative splicing affects neuronal development and function by regulating various proteins involved in synaptic connectivity and cellular excitation, including ion channels [2,3]. Voltage-gated ion channels control the flow of charged ions across the cell membrane and the resulting membrane potential fluctuations sculpt the excitability and overall firing patterns of the cell. Several mechanisms, including alternative splicing of channel transcripts, maintain a highly diversified population of ion channels needed to coordinate complex cellular activities and functions [4].

Calcium ion channel pore-forming α-subunits are encoded by ten genes, and of these, three express low-voltage-activated Ca_v_3.1/α1G, Ca_v_3.2/α1H, and Ca_v_3.3/α1I T-type channels [5]. T-type calcium channels open at low membrane potentials, regulate calcium influx, and trigger rebound action potential bursting following prolonged hyperpolarizations. These channels are widely expressed in the nervous system, the heart, and other tissues [6,7], and contribute to various physiological activities, suggesting the utility of a heterogeneous population of T-type channels. Extensive alternative splice isoform profiling of the human *CACNA1G*, *CACNA1H*, and *CACNA1I *genes demonstrates highly diversified T-type channel mRNA transcript populations with distinct electrophysiology properties [8-10]. The mouse *Cacna1g *gene encodes α1G channels ranging from ~240–260 kDa [11], and alternative splicing profiling of the orthologous human *CACNA1G *gene shows four cassette exons (14, 26, 34, and 35), two alternative splice donor sites (25C and 30B), four alternative splice acceptor sites (25A, 25A', 25A", and 31A), and one protein-coding intron (38B) [9,12].

To evaluate the expression patterns of the mouse *Cacna1g *gene regulated by alternative splicing, we profiled the *Cacna1g *mRNA using overlapping RT-PCR amplicons to confirm the orthologous human α1G splicing events and to identify potential mouse-specific splicing events. Compared to human α1G alternative splicing, six of the 11 splicing events are conserved in the mouse, and six additional splicing events appear exclusively in the mouse transcriptome. We have also identified two brain region-specific alternative splice patterns in the cerebellum. Our results further define the scope of alternative splicing of the mouse *Cacna1g *gene and provide potential evidence of tissue-specific mRNA transcripts in generating functionally distinct α1G T-type channel proteins.

## Results

### Increased *Cacna1g *mRNA Transcript Diversity

**Figure 1:**
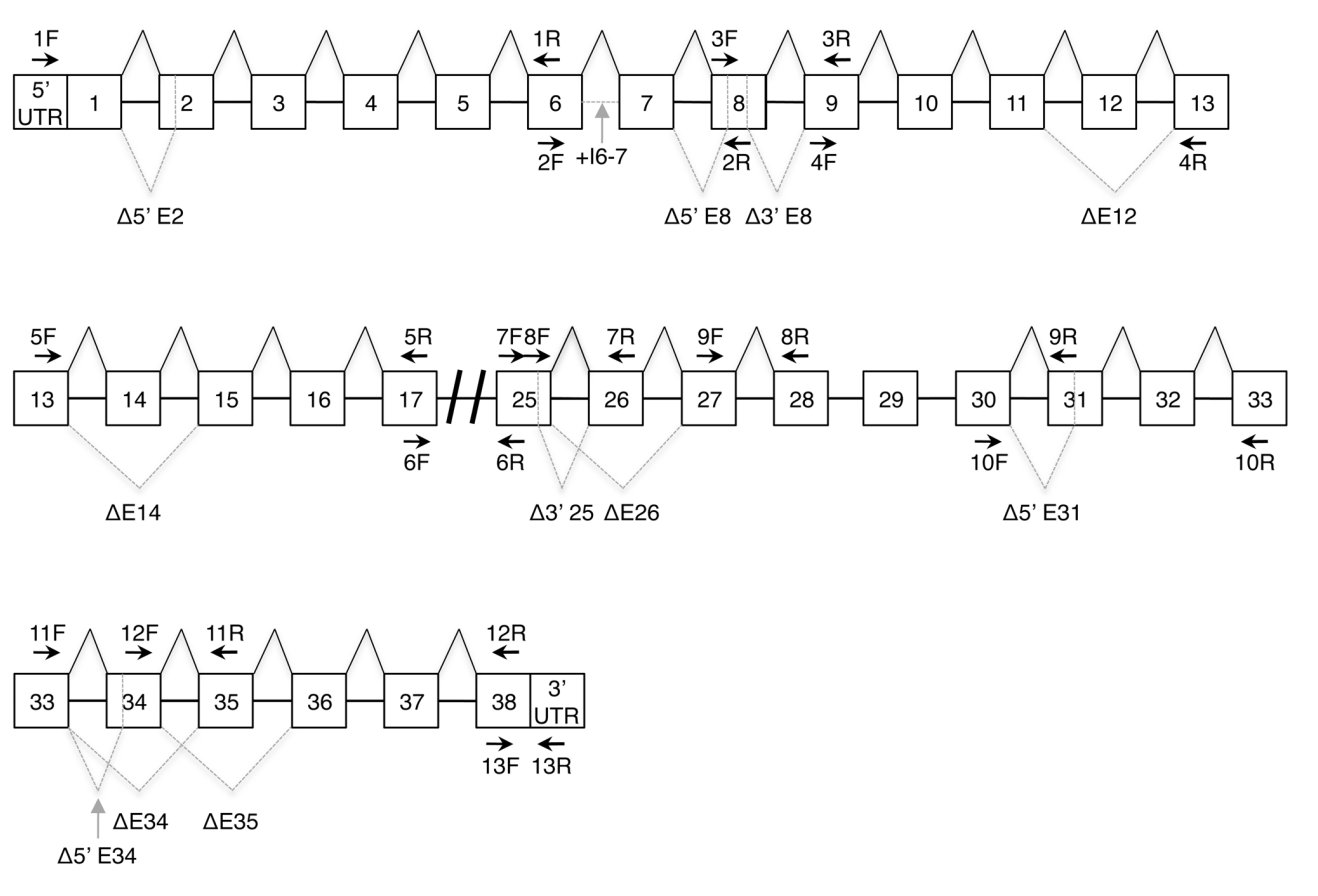
**Transcript scanning RT-PCR assay to detect alternative splicing events within the mouse *Cacna1g *gene**. Schematic representation of the mouse *Cacna1g *pre-mRNA sequence and the RT-PCR primers (arrows, see Table 1) used for transcript scanning. Dashed grey lines denote the alternative splicing events and alternative splice donor and acceptor sites. Intron and exon sequences are not drawn to scale.

**Figure 2:**
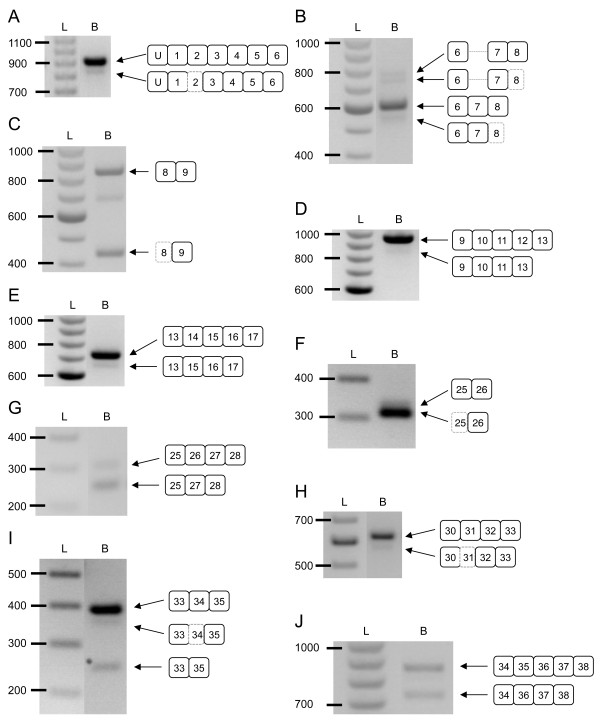
**Identification of the mouse *Cacna1g *alternative splicing events**. *Cacna1g *pre-mRNA undergoes extensive alternative splicing as detected by RT-PCR of mouse brain RNA samples. The spliced *Cacna1g *mRNA variants are illustrated to the right of each RT-PCR product: (A) Δ5' E2; (B) +I6-7 and Δ5' E8; (C) Δ3' E8; (D) ΔE12; (E) ΔE14; (F) Δ3' E25; (G) ΔE26; (H) Δ5' E31; (I) Δ5' E34 and ΔE34; and (J) ΔE35. Amplification of *Cacna1g *mRNA regions not undergoing alternative splicing was omitted. Lanes show the 100 bp ladder (L) and brain RT-PCR products (B). Intron and exon sequences are not drawn to scale.

To determine the alternative splicing sites and events occurring within the mouse brain *Cacna1g *mRNA population, we utilized a transcript scanning approach using overlapping RT-PCR amplicons to cover the full coding sequence (Table 1 and Figure 1). Using isolated brain mRNA samples, RT-PCR products representing alternative splicing variants were isolated, cloned, and sequenced (as detailed in the Methods). Six of the 11 potential orthologous human α1G alternative splicing events were verified in the mouse transcriptome. The common splicing events include four cassette exons (ΔE14, ΔE26, ΔE34, and ΔE35), one alternative splice donor site (Δ3' E25), and one alternative splice acceptor site (31A) [9] (Figures 1 and 2). We were unable to detect the other five alternative splice variants (25A, 25A', 25A", 30B, and 38B) in the mouse and these appear to be human-specific.

**Table 1:** RT-PCR primers for *Cacna1g *mRNA transcript scanning assay.

Primer and Sequence (5' → 3')		Exons	Splice Isoform(s)	Amplicon(s) (bp)
1F	CGGTTGTGTGAGGACACC	5' UTR→6	***Δ5' E2***	***852***, 900
1R	GAGGCTGAGAGCAGATGAAGG			

2F	AGACAGAGAATGAGGACGAGAGC	6→8	***+I6-7 ***&	***499***, 550,
2R	ATGTACACCAGGTACTTGAGAAGC		***Δ5' E8***	***664***, ***715***

3F	GTGTACGATTCCTGTCCAATGC	8→9	***Δ3' E8***	***428***, 830
3R	GTCTGAGTCAGGCATTTCATGG			

4F	CCATAGCTCCTGCAAAATCTCC	9→13	***ΔE12***	***826***, 951
4R	GCGACAAGCAGGTTAAAGAGC			

5F	CTCATGACTTTTGGCAACTACG	13→17	ΔE14 (e)^a^	623 (-e),
5R	TCAGGCAGGTCAAAGGAACT			692 (+e)

6F	GTCAGGAGAGCCAGGATGAG	17→25	None	1282
6R	AGCCTCTTTAGTCGCTTCTCC			

7F	GCTCTGATGTCCCTGTTTGTG	25→26	Δ3' E25	312 (b),
7R	GGAAGCAATTACATCGTCCAAC		(a/b)^a^	333 (a)

8F	TGGAGAAAAAGAGAAGGAGTAAGG	25→28	ΔE26 (c)^a^	238 (-c),
8R	ATGACGGTAAAGATGTAGTTGCAG			292 (+c)

9F	GATGGCCATGGAACATTAGG	27→31	None	371
9R	TGAAGAGAAGTCCCAGGTTCC			

10F	GCTGTTGAAGATGGCTGTGG	30→33	Δ5' E31^b^	561, 610
10R	AAGGGAGAAGCCTGAAGAGG			

11F	ACCTGGAAGAGAGCAACAAAGAG	33→35	ΔE34 (f)^a^/	256 (-f),
11R	GACAGAGCCTCCATCTCAGC		***Δ5' E34***	***361***, 400 (+f)

12F	AGACAGCTGTTTGACACCATCTC	34→38	ΔE35 (d)^a^	732 (-d),
12R	GCTGCTTCTGGTCTCTTGAGG			867 (+d)

13F	AGACAGCTGTTTGACACCATCTC	34→36	ΔE35 (d)^a^	215 (-d),
13R	CTGACACCAGACTTCCTCACAG			350 (+d)

14F	CTGACACCAGACTTCCTCACAG	35→38	None	656
14R	GCTGCTTCTGGTCTCTTGAGG			

15F	ACCTGTTGTCAGAGGTGAGTGG	38→3' UTR	None	718
15R	CCAGTGGAGAAAGGTGATGG			

Analysis of mouse *Cacna1g *mRNA transcripts by RT-PCR identifies 12 total alternative splicing events. The primer combinations (F, forward; R, reverse) are listed along with the scanned exons, the assayed splice isoforms, and the generated amplicon sizes. Newly-characterized splicing events and amplicon sizes are designated in bold, italic type. Orthologous mouse *Cacna1g *alternative splice isoforms are comparable to human *CACNA1G *splice sites, as previously identified by ^a^Mittman et al. (1999) and ^b^Emerick et al. (2006).

**Figure 3:**
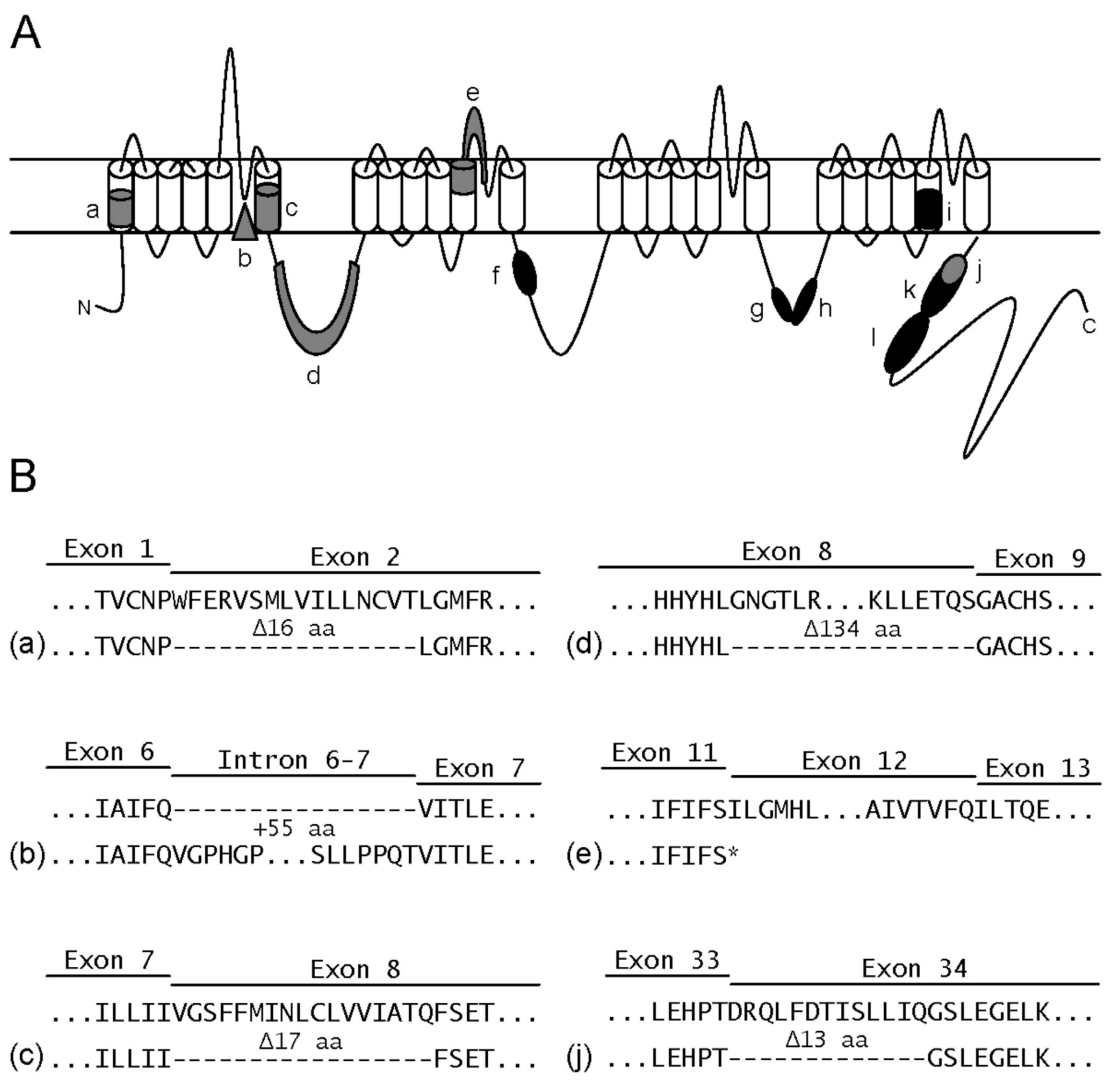
**Alternative splicing architecture of the mouse *Cacna1g *gene**. (A) Representation of the Ca_v_3.1/α1G T-type calcium channel structure and the localization of the 12 alternatively spliced regions: a) Δ5' E2; b) +I6-7; c) Δ5' E8; d) Δ3' E8; e) ΔE12; f) ΔE14; g) Δ3' E25; h) ΔE26; i) Δ5' E31; j) Δ5' E34; k) ΔE34; and l) ΔE35. Regions in black correspond to the previously identified alternative splice variants [9,12] and segments in grey denote the newly-characterized splice variants detected in this study. (B) Predicted protein sequences arising from the a), b), c), d), e), and j) alternative splicing events compared to full length Ca_v_3.1/α1G protein, dashes indicate in-frame amino acid exclusion and the asterisk signifies a nonsense codon truncation.

We confirmed six novel mouse-specific *Cacna1g *alternative splice variants, including one intron retention site (+I6-7), three alternative acceptor sites (Δ5' E2, Δ5' E8, and Δ5' E34), one alternative donor site (Δ3' E8), and one case of exon exclusion (ΔE12) (Figures 1 and 2). Use of the Δ5' E2 splice site causes skipping of the first 48 base pairs (bp) resulting in a predicted in-frame truncation of 16 amino acids (aa) in exon 2 (Figures 2A, 3A, and 3B). The alternatively spliced form localizes to the DI-S1 region of the channel. The second newly-identified splice variant, +I6-7, results in the retention of the intronic sequence between exons 6 and 7 (Figure 2B). Inclusion of this intron adds 165 bp to mRNA transcripts and a predicted 55 aa into the DI-P-loop (Figures 2B, 3A, and 3B). Exon 8 undergoes two independent alternative splicing events that utilize separate splice acceptor (Δ5' E8) and donor (Δ3' E8) sequences. The Δ5' E8 acceptor site signals splicing from the end of exon 7 into exon 8 after 51 bp, while the Δ5' E8 donor site initiates splicing into exon 9 402 bp before the end of exon 8 (Figures 2B and 2C). Both alternative splice sites generate in-frame truncations causing loss of the first 17 aa within DI-S6 (Δ5' E8) or the final 134 aa in the DI-II linker (Δ3' E8) (Figures 3A and 3B). The exclusion of exon 12 (ΔE12) in *Cacna1g *transcripts results in the loss of the entire 125 bp segment that normally encodes the end of the DII-S5 region and the beginning of the DII P-loop (Figures 2D, 3A, and 3B). However, alternative splicing produces an exon 11–13 junction that forms a novel mRNA sequence that prematurely adds a stop codon and truncates the channel protein. The final additional instance of alternative splicing occurs in exon 34, a previously characterized alternatively spliced exon that excludes the full sequence [12]. Exon 34 also contains an alternate splice acceptor site (Δ5' E34) that omits the first 39 bp of the exon (Figure 2I) and removes the 13 aa from the C-terminal tail (Figures 3A and 3B).

### Brain Region-Specific Alternative Splicing

**Figure 4:**
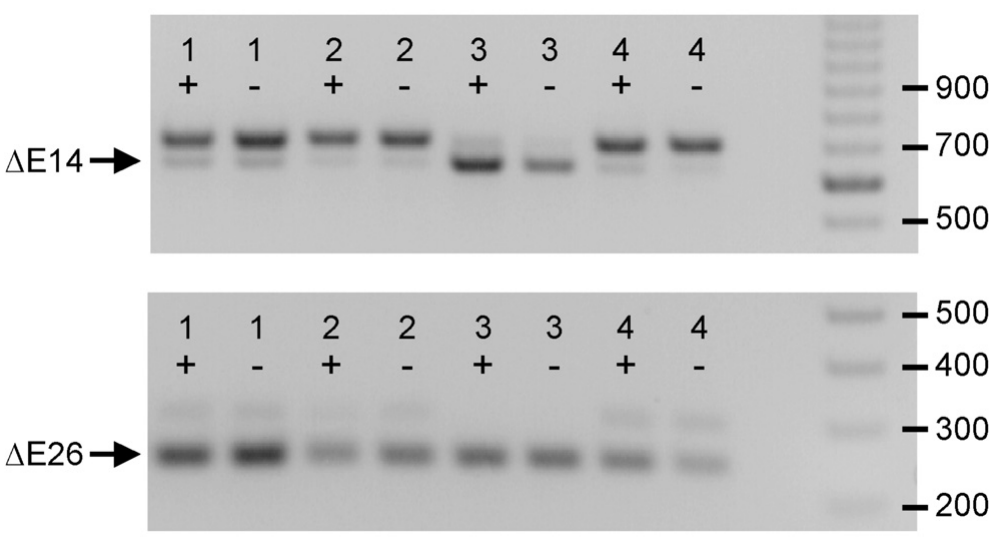
**Differential alternative splicing patterns detected in the adult mouse cerebellum**. RT-PCR reactions from dissected wildtype and *stargazer *brain region RNAs (lanes: 1, thalamus; 2, cortex; 3, cerebellum; 4, hippocampus; +, wildtype control; -, heterozygous mutant) reveal differential splicing patterns at exons 14 and 26 in the cerebellum. Amplification of exon 14 shows preference in expression for the alternatively spliced product, ΔE14, at 623 bp, over the typically spliced product at 692 bp. The amplification of exon 26 identifies a shift in splicing resulting in sole expression of the alternatively spliced product, ΔE26, at 238 bp, while the normally spliced product at 292 bp is absent. The DNA marker on the far right separates at 100 bp intervals. Dissected *lethargic *and *tottering *samples show similar splicing patterns (data not shown).

To assess the patterns of *Cacna1g *mRNA alternative splicing within distinct structures of the brain, we performed RT-PCR reactions on thalamus, cortex, cerebellum, and hippocampus RNA samples using the primer sets detailed in Table 1. Comparisons of the thalamic, cortical, and hippocampal samples revealed similar patterns of expression for each of the 12 alternative splice sites (data not shown). Analysis of splicing within the cerebellum of C57BL/6, *stargazer*, *lethargic*, and *tottering *mice identified two atypical patterns. The first occurred at exon 14, which encodes part of the DII-III linker, and results in elevated alternative splicing. As shown in whole brain (Figure 2E), the alternative splice product is normally the more weakly expressed variant, yet within the cerebellum, the ΔE14 splice isoform selectively predominated transcript expression. A second alteration of *Cacna1g *mRNA splicing is observed at exon 26, which encodes portions of the DIII-IV linker. In this instance, all transcripts lacked exon 26 due to exclusive alternative splicing in the cerebellum. Note the absence of the normally spliced product that includes exon 26 (Figure 4).

In some experiments, RT-PCR amplification of regions subject to alternative splicing produced hybrid, heteroduplex DNAs. These RT-PCR products were identified after cloning strategies failed, due to their altered structure. Alternative splicing at Δ3' E8 and ΔE14 generated intermediary heteroduplex dimers and digestion of purified RT-PCR products using the T7 Endonuclease I enzyme eliminated these DNA complexes (data not shown).

### Unaltered Alternative Splicing Patterns in Absence Epilepsy Mouse Models

**Figure 5:**
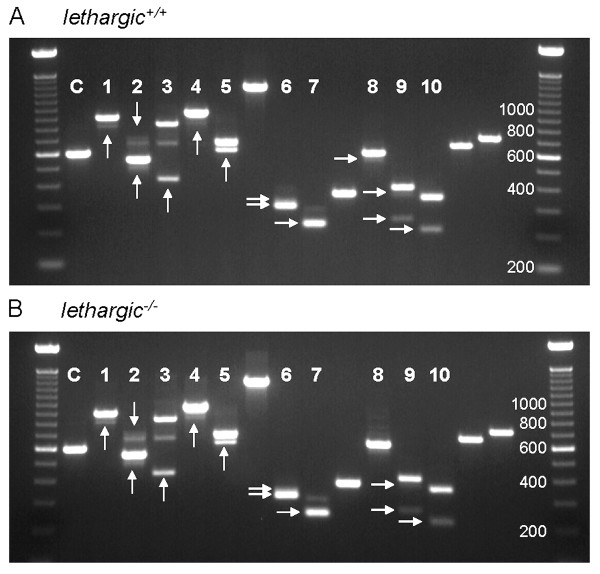
***lethargic *mutant mice display unaltered *Cacna1g *mRNA alternative splicing within the brain**. Compared to wildtype littermates (A), *lethargic *mutants (B) exhibit similar patterns of alternative splicing of *Cacna1g *transcripts assayed by RT-PCR from whole brain samples. Lanes within the agarose gel that are labelled (1–10) represent portions of the *Cacna1g *gene that undergo alternative splicing, while lanes without labels demonstrate regions of the gene not regulated by alternative splicing. Lane assignments: C, α-tubulin amplification loading control; 1, Δ5' E2; 2, +I6-7 & Δ5' E8; 3, Δ3' E8; 4, ΔE12; 5, ΔE14; 6, Δ3' E25; 7, ΔE26; 8, Δ5' E31; 9, Δ5' E34/ΔE34; 10, ΔE35. Alternative splice variants are denoted by the arrows.

**Figure 6:**
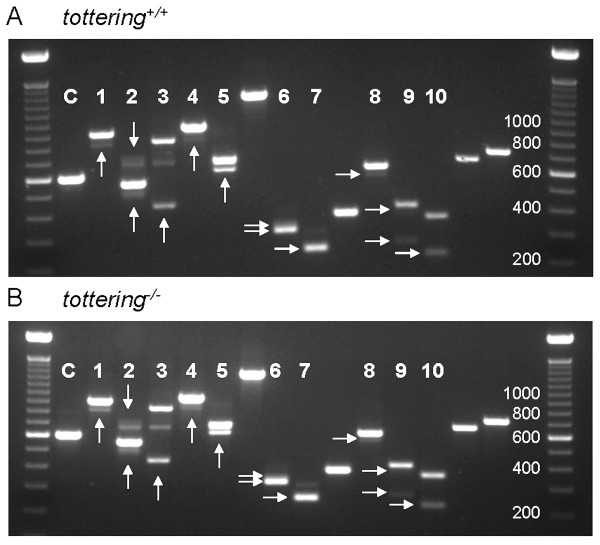
***tottering *mutant mice display unaltered *Cacna1g *mRNA alternative splicing within the brain**. Compared to wildtype littermates (A), *tottering *mutants (B) exhibit similar patterns of alternative splicing of *Cacna1g *transcripts assayed by RT-PCR from whole brain samples. Lanes within the agarose gel that are labelled (1–10) represent portions of the *Cacna1g *gene that undergo alternative splicing, while lanes without labels demonstrate regions of the gene not regulated by alternative splicing. Lane assignments: C, α-tubulin amplification loading control; 1, Δ5' E2; 2, +I6-7 & Δ5' E8; 3, Δ3' E8; 4, ΔE12; 5, ΔE14; 6, Δ3' E25; 7, ΔE26; 8, Δ5' E31; 9, Δ5' E34/ΔE34; 10, ΔE35. Alternative splice variants are denoted by the arrows.

Identification of the full profile of alternative splicing events in *Cacna1g *transcripts creates a valuable tool to assess the contribution of this regulatory mechanism to pathogenic T-type channel function in the mouse brain. We compared the splicing profiles within whole brain, cortex, thalamus, hippocampus, and cerebellum samples between wildtype and four absence epilepsy mutant mouse models previously shown in our laboratory to express elevated thalamic T-type currents, *lethargic*, *tottering*, *stargazer*, and *Coloboma *[13,14]. As presented in Figures 5 and 6, *lethargic *and *tottering *mutant mice show no apparent dysregulation of whole brain Ca_v_3.1/α1G channel alternative splicing in comparison to the wildtype pattern of expression. Similar results were observed within whole brain samples collected from *stargazer *and *Coloboma *mice (data not shown). Additionally, comparisons of dissected brain region samples showed identical splicing patterns between each mutant mouse and wildtype controls (data not shown).

## Discussion

The *lethargic*, *tottering*, *stargazer*, *Coloboma*, and *Cacna1a*^-/- ^mutant mouse models of absence epilepsy each exhibit significant increases in thalamic T-type calcium currents despite the absence of evidence for enhanced T-type channel subunit expression [13-15]. The cellular mechanism evoking this biophysical change remains unknown, but since T-type channels are subject to alternative splicing [8-10] and Ca_v_3.1/α1G channels distinctly localize to the thalamic relay nuclei where the enhanced T-type current densities are present [7], we reasoned that alternative splicing of T-type channels might underlie this pathogenic hyperexcitability phenotype.

In this study, complete alternative splice isoform scanning of the full open reading frame of the mouse *Cacna1g *gene revealed extensive molecular diversity of Ca_v_3.1/α1G T-type calcium channels. Previously, 11 alternative splice variants were identified within eight exons of the orthologous human *CACNA1G *gene [9]. Overlapping RT-PCR amplification of mouse *Cacna1g *transcripts identified six splicing events comparable to those known in human *CACNA1G*: ΔE14, Δ3' E25, ΔE26, Δ5' E31, ΔE34, and ΔE35. In addition, six novel mouse-specific splice variants were identified, resulting in 5' end truncations of exons 2, 8, and 34 (Δ5' E2, Δ5' E8, and Δ5' E34) through use of alternative splice acceptors, inclusion of the intron between exons 6 and 7 (+I6-7), 3' end truncation of exon 8 (3' E8) via utilization of an alternative splice donor, and exclusion of exon 12 (ΔE12). The 12 total splicing events in the mouse gene, occurring at 11 locations within *Cacna1g *mRNA transcripts, theoretically could generate 3072 (3 × 2^10^) distinct full-length transcripts. Two splice sites, ΔE12 and Δ5' E31, however, create modified exon-exon junctions that form premature nonsense stop codon sequences, thus reducing the pool of potential functional *Cacna1g *transcripts from 3072 to 768 (3 × 2^8^). Analysis of full-length human *CACNA1G *transcripts from fetal and adult brain samples identified only 30 total transcripts, 15 of which were distinct in fetal samples and 8 that are unique in adult samples [9]. In addition to the highly regulated developmental expression of transcripts, alternative splicing controls the systematic combinations of expressed exons [9]. These factors suggest that the vast number of potential combinations of alternatively spliced exons when forming complete transcripts is regulated to generate specific Ca_v_3.1/α1G T-type channel function. The developmental and activity-dependent control of these regulatory mechanisms in the mouse nervous system remain unknown and warrant further investigation. Nevertheless, the extensive potential diversity of mouse *Cacna1g *transcripts through alternative splicing may produce channel variants capable of redefining the functional identity of a cell.

Two of the newly-identified splice events localize to regions of T-type channels previously shown to alter channel kinetics. Alternative splicing at the mouse Δ3' E8 splice site truncates a large portion (134 aa) of the I-II linker domain. Splicing of this domain produces functional differences observed through the alternative splicing of human exon 9 in Ca_v_3.3/α1I T-type channels. The inclusion of this exon produced different effects dependent upon other splice variants. In the presence of full-length exon 33, channels exhibit a shifted voltage dependence of SSI and an increased recovery rate from inactivation near the resting potential, while alternative splicing of exon 33 increases the activation and inactivation rates at depolarized potentials [8]. A second related splicing event in the mouse occurs at exon 34 (Δ5' E34) that removes 13 aa encoding a portion of the C-terminal tail of the channel. Splicing at the C-terminus of Ca_v_3.3/α1I T-type channels also alters electrophysiological properties, as the spliced exon 33 produces slower activation and faster inactivation rates [8]. The observed kinetic changes induced by alternative splicing of Ca_v_3.3/α1I demonstrate that splicing at analogous sites of the Ca_v_3.1/α1G channels, Δ3' E8 and Δ5' E34, can promote altered channel kinetics.

Alternative splicing occurring at the Δ5' E2 and Δ5' E8 sites truncates the S1 and S6 transmembrane segments of domain I, respectively. Splicing within these transmembrane segments may perturb proper protein structure; however, the functional consequences of splicing within these regions are unknown. Furthermore, retention of the intronic sequence between exons 6 and 7 inserts 55 aa into the P-loop of domain I. While splicing does not affect the composition of the ion selectivity filter of the channel, expression of intron 6–7 may alter the physical structure of the pore by obstructing the alignment of the filter residues and therefore modify channel function. The identification of an additional six alternative splicing events, excluding the ΔE12 variant that generates a premature stop codon, now necessitates the cloning and expression of these transcripts, along with the electrophysiological analysis of their biophysical properties to fully comprehend their individual and combinatorial effects on Ca_v_3.1/α1G channel activity.

Expression profiling of the alternative splice patterns within the brain identified distinct regional splice variant expression within the cerebellum when compared to the thalamus, hippocampus, and neocortex. Cerebellar-specific splicing patterns observed due to the ΔE14 and ΔE26 splice variants result in preferential alternative splicing of exon 14 and exclusive splicing out of exon 26. Rebound burst firing activity in several cerebellar neurons arises in part from the coordinate expression of T-type calcium channel isoforms [16,17], and altered Ca_v_3.1/α1G channel function mediated by alternative splicing may initiate abnormal burst firing leading to movement disorders, such as the ataxia and paroxysmal dyskinesia phenotypes associated with absence epilepsy in several of these mutant mouse models.

Although several studies of absence epilepsy mutants have identified striking increases in thalamic T-type calcium currents, these elevations were not the result of clear increases in T-type channel expression [13-15]. Dysregulation of T-type *Cacna1g *channels induced from steady-state expression levels of T-type channels can account for the low-threshold burst firing associated with the absence epilepsy phenotype [18]. Specific Ca_v_3.1/α1G splice variants localize to various regions of the thalamocortical network [19,20]; therefore, comparative analysis of Ca_v_3.1/α1G splicing between mutant mice and wildtype controls was performed to determine whether this mechanism enhances T-type currents. The results of profiling alternative splicing of *Cacna1g *in the *tottering*, *lethargic*, *stargazer*, and *Coloboma *revealed unaltered splicing regulation of *Cacna1g *transcripts (Figure 5 and 6). This result indicates that even though extensive *Cacna1g *alternative splicing generates transcript variants that can enhance the Ca_v_3.1/α1G channel kinetics *in vitro *[9,10,21], *in vivo *splice isoform variation does not explain the elevated T-type currents in these models.

## Conclusion

In summary, 12 alternative splicing events found within the coding region of the mouse *Cacna1g *gene enhance the known diversity of T-type channel molecular structure. Although no alteration in splicing patterns of Ca_v_3.1/α1G channel mRNA was detected that could account for the elevated T-type currents linked to the generation of absence epilepsy in several known mouse mutant models, this mechanism may contribute to T-type calcium current variation in other developmental or pathological conditions.

## Methods

### Sample Collection

Tissue samples were collected from adult C57BL/6, *tottering*, *lethargic*, *stargazer*, and *Coloboma *mice and total RNA was extracted from whole brain samples and dissected brain regions (cortex, cerebellum, hippocampus, and thalamus) using TRIzol Reagent (Invitrogen) following the manufacturer's protocol. Mouse handling and experimental procedures were conducted in accordance with the guidelines of the US National Institutes of Health, as approved by the Institutional Animal Care and Use Committee of Baylor College of Medicine (BCM).

### Alternative Splicing RT-PCR Analysis

RNA samples were reverse transcribed using the SuperScript III First-Strand Synthesis System (Invitrogen) with random hexamer primers, according to the manufacturer's protocol, and primer sets (synthesized by IDT) spanning the entire mouse *Cacna1g *mRNA sequence [GenBank:NM_009783] were designed for PCR amplification (Table 1). Each PCR reaction contained 1.2× PCR Buffer, 2.5 mM MgCl_2_, 5% DMSO, 0.2 mM (each) dNTPs, 0.3 μM forward and reverse primers, 1.25 u Platinum *Taq *Polymerase, and 1.0 μl cDNA (PCR reagents purchased from Invitrogen). The thermal conditions consisted of an initial denaturation for 2 min at 95°C, 35 cycles of 94°C for 20 s/60°C for 30 s/72°C for 40 s, and a final extension for 7 min at 72°C, and RT-PCR products were resolved by agarose gel electrophoresis.

### Splice Variant Identification

Direct or gel extracted (QIAquick Gel Extraction Kit, Qiagen) RT-PCR products were TOPO cloned and transformed into One Shot TOP10 competent *E. coli *cells (Invitrogen), according to the manufacturer's protocol, and spread onto LB-agar plates. After an overnight incubation at 37°C, single colonies were first streaked onto a gridded LB-agar plate and then inoculated into 10 μl of sterile water. The bacterial suspension was heated for 10 min at 95°C to lyse the cells, cooled on ice, and added to a PCR reaction mix containing 1× PCR Buffer, 2.0 mM MgCl_2_, 0.2 mM (each) dNTPs, 0.5 μM forward and reverse primers, and 1.0 u *Taq *Polymerase (PCR reagents purchased from Invitrogen). Colony PCR was performed utilizing the thermal conditions used for the initial RT-PCR reaction. Positively screened colonies were then inoculated in LB media with antibiotics and grown overnight at 37°C. The plasmid DNA with RT-PCR product insert was isolated (QIAprep Spin Miniprep Kit, Qiagen) and sequence analyzed (BCM Child Health Research Center) to determine the *Cacna1g *mRNA splicing event.

RT-PCR reactions demonstrating the presence of alternative splice isoforms that were unable to be TOPO cloned were assayed for heteroduplex DNA formation. Double-stranded DNA products were purified using the QIAquick PCR Purification Kit (Qiagen), incubated with 5 u T7 Endonuclease I (New England Biolabs) at 37°C for one hour, and resolved by agarose gel (2% w/v) electrophoresis.

## Abbreviations

aa: amino acid(s); bp: base pair(s); Δ: deletion; LB: Luria-Bertani; PCR: Polymerase Chain Reaction; RT-PCR: Reverse Transcriptase Polymerase Chain Reaction; SSI: Steady-State Inactivation; u: units; UTR: Untranslated Region.

## Authors' contributions

WLE and JLN contributed to the design of this research project. WLE performed all experiments. WLE wrote the manuscript with assistance from JLN. All authors read and approved the final manuscript.
